# Dr. Evans on the Spread of the Cholera

**Published:** 1849-12

**Authors:** 


*Dr. Evans on the spread of the Cholera.*—This is a paper republished from the pages of the North Western Medical and Surgical Journal, by Prof. John Evans, one of the Editors of that print.

Prof. Dr. E. adduces facts connected with the spread of epidemic cholera in Chicago, to prove its communicability, or, in other words, the existence of a contagious principle by which the disease is diffused. We cannot concur with our friend in the conclusion at which he arrives, but we are free to admit that the facts which he adduces invests his hypothesis with much apparent plausibility. The author is an ardent as well as vigorous thinker, and displays, in the discussion of this subject, that ability which characterizes his editorial productions.

In contending for the contagiousness of cholera, he is not without coadjutors among the ablest and most eminent members of our profession, but an unbiased survey of all the facts bearing on this important question, as we are constrained to believe, must satisfy the majority of inquirers of the non-contagiousness of the disease.

